# Case report: Precise NGS and combined bevacizumab promote durable response in ALK-positive lung adenocarcinoma with multiple-line ALK-TKI resistance

**DOI:** 10.3389/fonc.2024.1419306

**Published:** 2024-06-24

**Authors:** Jin Xiong, Lei Xia

**Affiliations:** ^1^ Department of Cancer Center, The Second Affiliated Hospital of Chongqing Medical University, Chongqing, China; ^2^ Tianjin Key Laboratory of Radiation Medicine and Molecular Nuclear Medicine, Institute of Radiation Medicine, Chinese Academy of Medical Sciences and Peking Union Medical College, Tianjin, China; ^3^ Department of Radiation Oncology, Key Laboratory of Cancer Prevention and Therapy, Tianjin Medical University Cancer Institute and Hospital, National Clinical Research Center for Cancer, Tianjin’s Clinical Research Center for Cancer, Tianjin, China

**Keywords:** pleural effusion NGS, combination therapy, long-term survival, multiple-line ALK-TKI, case report

## Abstract

Liquid biopsies including pleural fluid or plasma are commonly applied for patients with advanced non-small cell lung cancer (NSCLC) and pleural effusion (PE) to guide the treatment. ALK-TKIs are the first options for patients with ALK-positive mutations and combining ALK-TKIs with angiogenic agents may improve survival. We report here one case with ALK-positive lung adenocarcinoma in which the patient achieved a prolonged progression-free survival (PFS) of 97 months after undergoing precise pleural effusion NGS and receiving combined bevacizumab treatment following multiple-line ALK-TKI resistance.

## Introduction

Inhibition of the kinase activity of anaplastic lymphoma kinase (ALK) by tyrosine kinase inhibitors (TKIs) has become the first line treatment for patients with advanced non-small cell lung cancer (NSCLC) harboring ALK-positive mutations (1). However, many patients eventually develop primary or acquired drug resistance, leading to disease progression. The mechanisms of acquired drug resistance are primary due to secondary mutations (2). Therefore, comprehensive genetic tests should be applied to detect arising resistance mechanisms after each progression, and the tumor tissue genetic test is widely used as a traditional method. However, liquid biopsies, with their easy accessibility, may replace or complement the tissue biopsies to identify newly acquired emerging resistance mechanisms in clinical practice (3).

Vascular endothelial growth factor (VEGF) plays a crucial role in the proliferation and metastases of tumors. Bevacizumab, a humanized monoclonal IgG1 antibody that binds to VEGF, has shown clinical efficacy in treating various types of malignant neoplasms. The synergistic application of TKIs and antiangiogenic agents such as bevacizumab in driver gene-positive patients is under exploration to further promote the patient survival. The BELIEF trial has proved the benefit of the combined use of erlotinib and bevacizumab in patients with NSCLC harboring activating EGFR mutations (4). Although the research on the combination of ALK-TKI and bevacizumab is limited, it is imperative to further recognize the efficacy of the combination therapy in patients with ALK-positive mutations to guide clinical decision-making. Herein, we present a case of ALK-positive lung adenocarcinoma in which the patient achieved extended survival after undergoing precise pleural effusion NGS and combined bevacizumab treatment following multiple-line ALK-TKI resistance.

## Case description

**Figure 1:**
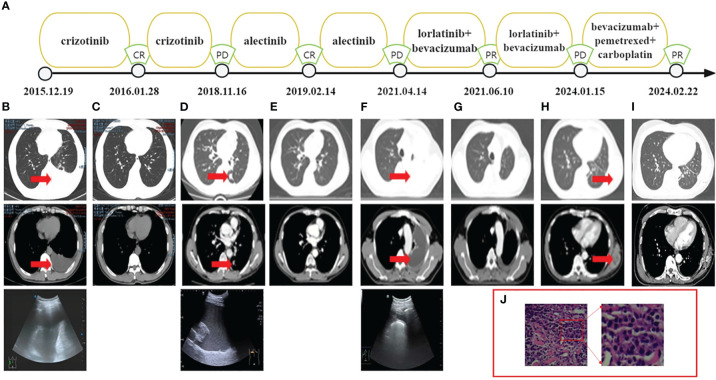
**(A)** Treatment flow chart. **(B)** CT and ultrasound showed the left pleural effusion. **(C)** CT indicated CR. **(D)** CT showed a lower lobe nodule of the left lung with a maximum length of 1.3 cm and ultrasound showed the left pleural effusion. **(E)** CT indicated CR. **(F)** CT and ultrasound showed the left pleural effusion. **(G)** CT indicated PR. **(H)** CT showed the mass on his left posterior chest wall. **(I)** CT indicated PR. **(J)** H&E, hematoxylin and eosin stain of the metastatic mass. CT, computed tomography.

In December 2015, a 57-year-old male presenting with chest pain and post-activity fatigue was diagnosed with left lower lung adenocarcinoma (cT1N2M1a stage IVA) upon identification of cancer cells in his left pleural effusion (**Figure 1B**). The NGS of the paraffin-embedded section from pleural effusion revealed an ALK-positive mutation (EML4-ALK fusion). Consequently, the patient began self-administering crizotinib from 30th December 2015, at a dose of 250 mg twice daily. An initial complete response (CR) was observed by 1th January 2016 (**Figure 1C**) and bi-monthly follow-ups showed no drug related adverse effects including rash or hypertension. The patient exhibited a PFS1 of 35 months.

On 16th November 2018, the patient underwent chest ultrasound scan for post-activity fatigue, which indicated massive left pleural effusion. The subsequent pleural effusion cytological examination found cancer cells. Meanwhile, computed tomography (CT) showed a lower lobe nodule of the left lung with a maximum length of 1.3 centimeters (**Figure 1D**). The efficacy assessment for the patient was progression disease (PD). No ALK related mutations were detected by circulating tumor DNA sequencing from the plasma sample. Nevertheless, DNA sequencing of paraffin-embedded section from pleural effusion revealed the ALK point mutation (F1174C exon23) on Illumina high-throughput sequencing platform. The patient was then switched from crizotinib to alectinib at a dose of 600 mg twice daily since 4th December 2018. On 14th February 2019, the patient achieved CR. (**Figure 1E**) and exhibited a PFS2 of 29 months.

On 14th April 2021, an ultrasound scan and cytological examination confirmed recurrent massive malignant left pleural effusion (**Figure 1F**). A second PD was considered for the patient and follow-up circulating tumor DNA sequencing of the pleural fluid identified two ALK point mutations (F1174C exon23, V1180L exon23) on Illumina high-throughput sequencing platform. The patient was then administered the treatment of lorlatinib (100mg once daily). In order to prolong the PFS for the patient, bevacizumab was concurrently applied to the patient at a dose of 400 mg every three months since 28th April 2021. The patient achieved a partial response (PR) on 10th June 2021 (**Figure 1G**) and exhibited a PFS3 of 33 months.

On 15th January 2024, the patient presented at hospital due to a mass he found on his left posterior chest wall (**Figure 1H**). A percutaneous aspiration biopsy of this mass confirmed metastases (**Figure 1J**) and excluded the possibility of pathological transformation such as the squamous cell carcinoma or small cell carcinoma. The metastatic tissue underwent NGS on Illumina high-throughput sequencing platform and revealed the intergenic-ALK rearrangement. Radioactive iodine125 particles implantation surgery was performed on the local lesion and the patient received systematic treatment consisting of bevacizumab (400mg), pemetrexed (800mg) and carboplatin (450mg AUC=4) every three weeks.

On 22th January 2024, the CT revealed that the patient had achieved a PR after one cycle of chemotherapy and local radioactive therapy (**Figure 1I**). During the whole series of treatment, no obvious drug-related adverse effects, such as rash, fatigue, hypertension and hypertriglyceridemia were observed in the patient. All the NGS conducted on the Illumina high-throughput sequencing platform were performed with the equipment of Illumina NovaSeq6000 platform (Illumina, SanDiego, CA) based on the Yuansu706 gene panel or Xiyuan18 gene ctDNA panel (OrigiMed, Shanghai, China). Treatment flow chart is presented below (**Figure 1A**) and the ALK-related genetic tests results are shown in **Table 1**.

**Table 1:** Genetic tests of patient during the treatment.

Date	Sample (Number)	Tumor Response	ALK Variations (frequency)	Co-mutations (frequency)
2015/12/30	paraffin section made from pleural effusion	Baseline	EML4-ALK E6:A20	None
2018/11/24	blood sample (CP60018920)	PD after 35months of crizotinib	None	None
2018/11/24	paraffin section made from pleural effusion	PD after 35months of crizotinib	ALK F1174C exon23 (26.6%) ALK intergenic rearrangement 2p11.1; ALK exon 20-29	CDKN2A deficiency CDKN2B deficiency TP53 E204* exon6 & R273H exon8 (3.8%) KDM5C K935* exon19 (0.7%)
2021/04/05	pleural effusion	PD after 29months of alectinib	ALK F1174C exon23 (24.4%) ALK V1180L exon23 (23.9%) ALK intergenic rearrangement 2p11.1; ALK exon 20-29	CDKN2A deficiency RB1 R255* exon8 (0.1%)
2024/01/24	lesion tissue from the left posterior chest wall	PD after 33months of bevacizumab and lorlatinib	ALK intergenic rearrangement 2p11.1; ALK exon 20-29	CDKN2A deficiency CDKN2B deficiency MTAP deficiency CHD4 R1162W exon24 (1%) TP53 M340Tfs*4 exon10 (41%)

PD, progression of disease; EML4, echinoderm micro tubule-associated protein 4; ALK, anaplastic lymphoma kinase.

## Discussion

### Pleural effusion offers potential therapeutic opportunities

In our case, the NGS results of the blood extracted from the patient were ALK-negative after the drug resistance of crizotinib yet the DNA sequencing of paraffin-embedded sections from the pleural effusion revealed an ALK point mutation (F1174C exon23). The different results of genetic tests between the plasma and pleural effusion samples should evoke our thought into the comparison of the two types of materials. Pleural fluid and plasma biopsies appeared to be the only available samples for many patients with advanced malignancies accompanying with pleural effusion (5). One study has confirmed that PE-cfDNA has a higher sensitivity for mutation detection than plasma cfDNA in hemorrhagic or cytologically negative PE samples, and genomic profiling of PE-cfDNA offers an alternative, more meticulous approach to assess tumor genomics in advanced lung cancer when tumor tissue is not available (6). Another study also proved that PE supernatant had higher sensitivity than plasma for identifying actionable mutations including resistance mutations (7). In our case, the patient had the opportunity to restart the ALK-TKI therapy based on the molecular detection of PE rather than prematurely turning to chemotherapy after the failure of plasma sample, which may have otherwise severely impaired his survival. Therefore, the supernatant or paraffin-embedded sections from malignant pleural effusion may be indispensable options for patients with malignant pleural effusion who are unable to acquire lesion tissue for genetic testing.

### Prompt assessment and lorlatinib prevent CNS progression effectively

Meanwhile, we observed a particular phenomenon that the patient repeatedly presented with malignant PE during the course of disease, which required careful assessment as potential PD in clinical practice. The response evaluation criteria in solid tumors (RECIST 1.1) are widely applied for the assessment of the efficacy in solid tumors and the malignant PE is deemed as non-measurable lesions. It was difficult to identify whether the patient could be confirmed as PD when only the malignant PE progressed, but PD could be confirmed given that the malignant PE turned from a small to a large amount (8). It was crucial for us to make prompt and accurate judgment in clinical practice so that the patient could benefit from sequential therapies and avoid metastases to other important organs including brain, live or bones. Patients with ALK-positive NSCLC are more likely to develop brain metastases than patients with RET or ROS1 rearranged NSCLC, with a cumulative incidence of 60% at 6 years (9). However, the patient in our report did not develop the brain metastases during the course and we speculated that lorlatinib had also played an important role as it was highly effective at preventing CNS progression in the majority of patients. The 12-month cumulative incidence rate of CNS progression in patients with or without brain metastases at baseline was only 7% and 1% respectively in CROWN study (10). Therefore, the patient in our study may benefit from lorlatinib and thus presented long-term intracranial PFS.

In the latest assessment of PD, the mass on the patient’s left posterior chest wall was pathologically confirmed as metastases. We speculated that it might be caused by the thoracentesis as the relapse site was located in the area where the puncture needle crossed. The incidence of tumor implantation metastases in the needle tract is low and there are different reports in the literature regarding the incidence of tumor needle tract implantation metastases. Kosugi et al. (11) found the incidence of tumor needle tract implantation was 1.6%-1.4% and the average time from puncture to tumor detection was 11.2 months (5-25 months). Chang et al. (12) reported that the incidence of tumor needle tract implantation was 0.76% and the average time from puncture to detection of tumor implantation was 9 months (4-21 months). The following measures could be taken to kill the residual tumor cells in the needle tract: standardization of operation procedures; reduction of unnecessary punctures; injections of anhydrous alcohol or chemotherapeutic drugs into the puncture tracts; soaking the implanted needles with chemotherapeutic drugs (13). This case we reported reminded us that skillful operations and appropriate preventive measures should be taken to prevent tumor needle tract implantation. Meanwhile, regular follow-up may guide early detection and treatment.

### Standard and precise change of ALK-TKIs benefit patient

Different generations of ALK-TKIs present inconsistent sensitivity to patients with various ALK-related mutations, therefore, a second NGS was entailed to guide the subsequential therapeutical decisions once progression of disease or drug resistance were confirmed. During the treatment of the patient, EML4-ALK fusion, ALK F1174C and V1180L point mutations were discovered and they present different functions and characteristics. ALK gene fusions, the majority of which are EML4-ALK fusions caused by chromosomal translocations, are present in 2-7% of NSCLC patients (14). Crizotinib, ceritinib, alectinib, brigatinib and lorlatinib have been approved by FDA for the treatment of patients with ALK fusion-positive metastatic NSCLC. ALK F1174C mutation is a substitution of phenylalanine for cysteine at position 1174 of the ALK gene, which is considered a secondary mutation that is crizotinib- and ceritinib-resistant but alectinib-sensitive (15, 16). ALK V1180L mutation is a change from valine to leucine at codon 1180 of the ALK gene, which is located in the structural domain of the ALK kinase. This ALK point mutation is discovered in the alectinib-acquired-resistant NSCLC H3122 cell line and it has been proven to be resistant to crizotinib and alectinib but sensitive to ceritinib (17). Preclinical studies have shown that lorlatinib could inhibit cell proliferation and induce apoptosis in NSCLC cells that harbor the EML4-ALK fusion and the V1180L mutation through inhibition of AKT phosphorylation (18). The patient had undergone several standard and correct changes of ALK-TKIs during the treatment based on precise NGS, which had played significant role in promoting the survival. Therefore, it is crucial to choose the appropriate ALK-TKIs when patients develop drug resistance to archive better survival outcome.

### Combination treatments may improve survival

After the patient developed resistance of alectinib, a combination therapy consisting of bevacizumab and lorlatinib was applied. We observed a surprisingly long survival benefit for the patient with a PFS3 of 33 months which was even longer than the PFS2. Lorlatinib is now widely applied as first line treatment for patients with ALK positive NSCLC. With a 3-year PFS rate of 63.5%, the results from the CROWN study indicated that lorlatinib has the potential to ultimately exceed 60 months in median PFS as a first-line treatment in patients with ALK positive NSCLC (19). However, prospective clinical studies of lorlatinib as a third line treatment are limited. One study found that Chinese patients with ALK-positive NSCLC who were previously treated by ALK-TKIs archived a median PFS of 5.6 months after receiving lorlatinib (20). Another study retrospectively analyzed patients with advanced ALK-positive NSCLC treated with lorlatinib and demonstrated that the median TTF for the third or back line treatment was 11.5 months (21). We speculated that bevacizumab had played an important role in prolonging the PFS3 of the patient. Studies have shown that bevacizumab, as a first-line drug in combination with conventional chemotherapy, targeted therapy and immunotherapy, can significantly improve PFS and life expectancy (22). Several clinical cases have shown that combining bevacizumab with lorlatinib successfully delayed the progression of disease and improved the quality of life in patients with ALK-positive NSCLC after undergoing multiple drug resistances (23). One recent ongoing single arm open-label phase II study (ClinicalTrials.gov identifier NCT03779191) revealed that the 2-year PFS rate was 80.8% and ORR was 100% in 41 patients receiving alectinib combined with bevacizumab, suggesting that antiangiogenic therapy may delay the incidence of drug resistance and enhance the efficacy of ALK-TKIs. Another phase I/II trial (ClinicalTrials.gov identifier NCT02521051) found that the treatment of alectinib plus bevacizumab was well tolerated without unanticipated toxicities or dose-limiting toxicities in patients with advanced ALK-rearranged NSCLC (24). The detailed information of the two clinical trials is shown in **Table 2**. The mechanism by which bevacizumab enhances the anti-tumor effects of alectinib has not been fully elucidated. Watanabe et al. (25) showed that VEGF-A and VEGFR-2 are highly expressed in ALK-altered cell lineage. They found that the blockade of VEGFR2 suppressed the proliferation of ALK-altered cells by inhibiting the oncogenic signaling pathway *in vitro*. Therefore, combined treatment with ALK-TKIs and bevacizumab may circumvent drug resistance and show promising efficacy as well as tolerable safety in clinical practice.

**Table 2:** Study characteristics of included clinical trials.

Author, Year, NCT number	Population (No. of patients)	Treatment	PFS/ORR	AEs reporting rate
**Oscar G Arrieta Rodriguez et al., 2018, NCT03779191**	Untreated and previously treated advanced ALK-rearranged non-squamous NSCLC (44)	Alectinib 600mg bid and bevacizumab 15 mg/kg every 3 weeks	mPFS: NR 2-year PFS rate: 80.8% ORR: 100%	Grade≥3 AEs: 51.2%
**Justin Gainor et al., 2015, NCT02521051**	Advanced ALK-rearranged non-squamous NSCLC (11)	Alectinib 600mg bid and bevacizumab 15 mg/kg every 3 weeks	mPFS:19.1 months ORR:81.8%	Grade≥3 AEs: 27.3%

NCT, national clinical trial; NSCLC, non-small cell lung cancer; PFS, progression-free survival; ORR, objective response rate; NR, not reported; AEs, adverse events.

## Conclusion

This study documents the effective treatment of a patient with lung adenocarcinoma accompanied by ALK-positive mutations, who exhibited successive resistance to crizotinib, alectinib and lorlatinib. This finding has influenced clinical practice, highlighting the value of reassessing patients for genetic mutations using pleural effusion and plasma to offer potential therapeutic opportunities. Meanwhile, combination therapy of antiangiogenic agents and ALK-TKIs may provide survival benefits for patients with multiple-line ALK-TKI resistance. This study underscores the needs for further genetic tests and individualized treatment strategies for such cases in clinical practice.

## Data availability statement

The original contributions presented in the study are included in the article/supplementary material. Further inquiries can be directed to the corresponding author.

## Ethics statement

Written informed consent was obtained from the individual(s) for the publication of any potentially identifiable images or data included in this article.

## Author contributions

JX: Writing – original draft, Software, Methodology, Investigation, Data curation, Writing – review & editing, Formal analysis. LX: Validation, Supervision, Conceptualization, Writing – review & editing, Formal analysis.
